# Geosynchronous Satellite GF-4 Observations of Chlorophyll-a Distribution Details in the Bohai Sea, China

**DOI:** 10.3390/s20195471

**Published:** 2020-09-24

**Authors:** Lina Cai, Juan Bu, Danling Tang, Minrui Zhou, Ru Yao, Shuyi Huang

**Affiliations:** 1 Marine Science and Technology College, Zhejiang Ocean University, Zhoushan 316004, China; clnown@zjou.edu.cn (L.C.); 16621727803@163.com (J.B.); 15156255226@163.com (M.Z.); dayaodaru@163.com (R.Y.); ehuangshuyi@163.com (S.H.); 2 Southern Marine Science and Engineering Guangdong Laboratory Guangdong Key Laboratory of Ocean Remote Sensing (LORS), South China Sea Institute of Oceanology, Chinese Academy of Sciences, Guangzhou 510301, China

**Keywords:** GF-4, chlorophyll-a, Bohai Sea

## Abstract

We analyzed the distribution of chlorophyll-a (Chla) in the Bohai Sea area based on data from the geosynchronous orbit optical satellite Gaofen-4 (GF-4), which was launched in 2015, carrying a panchromatic multispectral sensor (PMS). This is the first time the geosynchronous orbit optical satellite GF-4 remote-sensing data has been used in China to detect the Chla change details in the Bohai Sea. A new GF-4 retrieved model was established based on the relationship between in situ Chla value and the reflectance combination of 2 and 4 bands, with the R^2^ of 0.9685 and the total average relative error of 37.42%. Twenty PMS images obtained from 2017 to 2019 were applied to analyze Chla in Bohai sea. The results show that: (1) the new built Chla inversion model PMS-1 for the GF-4 PMS sensor can extract Chla distribution details in the Bohai Sea well. The high Chla content in the Bohai Sea is mainly located in coastal areas, such as the top of Laizhou Bay, Bohai Bay and Liaodong Bay, with the value being around 13 µg/L. The concentration of Chla in the Bohai Strait and northern Yellow Sea is relatively low with the value being around 5 µg/L. (2). Taking full advantage of the continuous observation of geostationary orbit satellite, GF-4 with a high-resolution sensor PMS of 50 m can effectively detect short-term change (changes within 10 min) in Chla concentration. The changes mainly appear at the southwest and northeast costal area as well as in the center of Bohai Sea with the change value of around 3 µg/L. (3) The change of Chla concentration in the Bohai sea is related to the environmental factors such as seawater temperature, salinity, illumination and nutrient salts, as well as the dynamic factors such as wind, flow field and tidal current.

## 1. Introduction

The Bohai Sea, located in north eastern China, is a semi-closed shallow sea, into which a large amount of sewage is discharged along the Gulf Coast. Environmental pollution of the Bohai Sea is becoming increasingly serious. The eutrophication of the water body is significantly intensified, resulting in frequent occurrence of red tides [1].

As an important part of marine environmental factors and water quality parameters, chlorophyll-a (Chla) has great significance for the marine ecological environment [2]. The detection of Chla concentration based on remote-sensing technology aims to calculate the Chla concentration using off-water reflectance [3] after atmospheric correction [4] and geometric correction of remote-sensing satellite image data. In prior studies, different inversion models [5] are developed to retrieve Chla concentration according to the off-water emissivity.

For the typical type II water body in the Bohai Sea, it is an urgent task to monitor the water color and quality change details of the sea [6]. Chla concentration can help us monitor the water quality and understand the characteristics of the coastal waters, as well as provide scientific basis for the management, and comprehensive utilization of the Bohai Sea [7,8,9]. It will be significant to find a more suitable Bohai Sea algorithm of Chla concentration inversion for the new sensor. The inversion algorithms for detecting Chla based on remote-sensing technology can be divided into two categories: empirical formula methods and model-based analytical methods [10]. At the beginning of 1990, Gitelson calculated the Chla concentration based on the ratio of the red band and the infrared band [11]. Meanwhile, neural network and semi-analytical algorithms, as well as bio-optical models were also used to invert Chla concentration [12]. Shu Xiaozhou adopted Gitelson’s method, corrected by using the phycoerythrin absorbance in 624 nm spectra, to improve the calculation accuracy of Chla [13]. In China, many prior studies analyzed the correlation [14,15] between the normalized difference and the in situ Chla concentration, and found that the two show a significant negative correlation. The Chla in type II waters retrieved from the incineration algorithm shows that the average absolute error of the results is 1.081 [16]. Using satellite remote-sensing data, the detection of Chla concentration of type II water is greatly affected by suspended sediment, colored dissolved organic matter (CDOM), etc. [17]. A multi-factor algorithm was established previously by researchers for ocean water color hyperspectral information. The quantitative relationship between the common contributions of water color factors such as Chla, suspended sediment, and CDOM and the emissivity of water was developed based on high-resolution and hyperspectral remote-sensing fusion data [18]. At present, there are many empirical and semi-empirical algorithms for marine Chla inversion, and most of them use satellite data, such as Moderate-resolution Imaging Spectroradiometer (MODIS) data and Operational Land Imager (OLI) data [19,20,21,22], from the near-polar solar synchronous orbit.

In recent years, due to the influence of manmade and natural factors, the environment of China’s coastal waters has undergone great changes, and the nutrient salt structure of some sea areas has changed, resulting in increasingly frequent red tide disasters [23]. Prior studies found that, in general, the annual variation of Chla concentration in the far coastal waters of Bohai bay is relatively low. Furthermore, the Chla concentration variation is characterized by a large range of continuous changes, and the seasonal variation in the near shore waters is obvious due to the influence of current conditions and precipitation [2]. Most important of all, the effect of tidal wave motion on Chla concentration distribution cannot be ignored. Tidal wave movement is the most important marine dynamic process in the Bohai Sea and plays an important role in controlling the marine environment [24].

The Gaofen-4 (GF-4) satellite is a geosynchronous orbit optical satellite with an orbital height of 36,000 km and a high resolution of 50 m, which has the advantages of realizing real-time cryptic observation of the marine environment [25,26]. GF-4 can observe disaster events such as algal blooms and red tides through pointing control to provide fast and reliable optical remote-sensing data [27]. The GF-4 satellite is the Earth observation satellite with the highest geostationary orbit resolution in the world. The electronic maritime surveillance satellite constellation basically meets the needs of ship surveillance in terms of coverage and time resolution [28]. It not only has continuous surveillance capabilities and large-scale coverage capability, but also has a geostationary orbit (GEO) high-resolution optical detection with fast response capability and higher positioning accuracy (better than 200 m) [29]. It can realize continuous monitoring of key targets, and make up for the problems of low time resolution and small coverage of traditional reconnaissance satellites. Meanwhile, it will also launch a series of new application areas, including the ability to move across the surface of the Earth based on video shooting methods, target detection and monitoring [30], long-term evolution monitoring of various natural elements of the Earth, etc. Furthermore, it has a quick response capability, and the delivery time from user’s application to geospatial information can be reduced to a few minutes. GEO high-resolution optical imaging satellite is a new generation of optical remote-sensing satellite [31]. Its outstanding features can be summarized in two words, one is “moving target surveillance” and the other is “real-time surveillance”. It raises the satellite reconnaissance function to the surveillance function, which can realize the dramatic change of satellite application. In addition, GEO high-score satellites can enhance the value of information applications based on new product types [29]. However, there is no suitable empirical model based on GF-4, a high-resolution satellite in geosynchronous orbit, for Chla inversion in the coastal waters such as Bohai Sea. Therefore, it is urgent for us to develop Chla inversion algorithms for GF-4.

In this paper, the concentration distribution and change details of Chla is analyzed based on the panchromatic multispectral sensor (PMS) data of the newly launched high-resolution GF-4 satellite in synchronous orbit. Based on GF-4 satellite PMS data and field measurement data, an empirical algorithm model of Chla inversion is established. The advantages of geostationary orbit satellite for continuous observation are used to analyze the detailed change of Chla in a short time (within 10 min) in the Bohai Sea.

The structure of this paper is organized as follows. Section 2 describes the study area, satellite date and data processing. Section 3 describes the detailed process of establishing the inversion model of Chla concentration. The discussion and conclusions are summarized in Section 4 and Section 5.

## 2. Data and Method

### 2.1. Study Area

**Figure 1:**
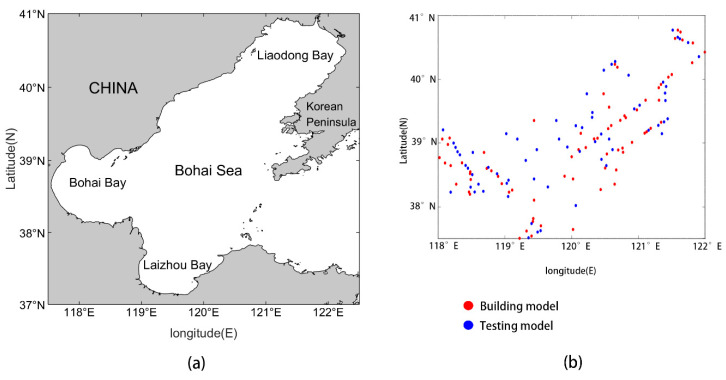
Location of Bohai Sea. (**a**) Study area; (**b**) the distribution of sampling points in study area, in which red points represent the sampling points of building model and blue points represent the sampling points of the testing model.

The Bohai Sea (37°07′~40°56′ N, 117°33′~122°08′ E) is an inland sea in China (Figure 1a). Surrounded by the three sides and communicating with the Yellow Sea through the Strait, the Bohai Seaport is 59 nautical miles wide and contains more than 30 islands [32]. As an important spawning ground and a bait fattening farm for China’s economy fish and shrimp, 40% of the important fishery resources in the Yellow Sea, Bohai Sea and the northern East China Sea are spawning here [2]. In recent years, however, fishery resources have been seriously declining, especially for high-quality species. The marine Chla is a crucial indicator of the existing amount of phytoplankton, and primary productivity [33]. Therefore, Chla and primary productivity are very important in the Bohai Sea investigation of the environment and resources. The detailed information of study area and sampling points is shown in Figure 1.

### 2.2. Satellite Data

The GF-4 satellite is a geosynchronous orbit optical satellite with an orbital height of 36,000 km and a resolution of 50 m, which fills the gap of high-resolution geosynchronous orbit optical remote-sensing satellites in China. GF-4 was successfully launched at the XiChang Satellite Launch Center on 29 December 2015, and sent back the first image on 5 January 2016 [34]. This is China’s first high-resolution geosynchronous orbit remote-sensing satellite, equipped with six bands and a visible light staring camera (PMS) with a resolution of 50 m/mid-wave infrared 400 m and a width of more than 400 km (Table 1) [35]. The GF-4 satellite provides fast, reliable and stable optical remote-sensing data for disaster detection, coastal zone management, meteorology and other applications, opening up a new field of high-resolution Earth observation in geosynchronous orbit [36]. In this study, 20 PMS images from GF-4 were applied to analyze Chla concentration and distribution trend in Bohai sea. The detailed information about the PMS images including the bands, resolution, width and revisit time of the PMS sensor is shown in the Table 1.

### 2.3. In Situ Data

The data sampling activities were conducted in the Bohai Sea from 146 stations as shown in Figure 1b at around three o’clock in the afternoon on 2 September 2019. We drew seawater in the ocean at around 5 m depth using the water pump, allowing seawater to enter the sample chamber. In this way we could determine the content of Chla in phytoplankton in the seawater. In total, 146 Chla samples were collected in study area (Figure 1a) for measurements. The in situ samples are first randomly divided into a modeling group and a testing group, which are 78 and 68 samples, respectively.

Chla data were obtained through the following steps: (1) filtration: 100–500 mL water samples were filtered with a fiberglass filter and we recorded the volume of the filtered water samples. We rolled the filter paper into a cigarette shape and placed it in a vial or centrifuge tube. We soaked the filter paper with 90% acetone solution, recorded the volume, plugged the plug, and left it in darkness at 4 °C for 4 h. (2) Extract: a survey of Chla fluorescence extraction method was used to extract: Chla of acetone extract of red blue light excitation fluorescence, filtering, a certain volume of water (mainly filter is the phytoplankton), with 90% acetone extract pigment, the use of fluorometer determination to extract fluorescence value before and after acidizing, and calculation of the concentration of Chla. In addition to the extraction fluorescence method, spectrophotometry and high-performance liquid chromatography (HPLC) method can also be used for determination. Two parallel samples were set for all the samples, and the mean value of the two measurements was taken as the final concentration [37].

### 2.4. Data Processing

#### 2.4.1. Radiation Calibration

In this paper, pretreatments such as radiation calibration, atmospheric correction, were performed on the L1A-level GF4_PMS multispectral data (http://dds.nsoas.org.cn/mainIndex.do) in the Bohai Sea on 2 September 2019 to obtain true reflectance [38].

Atmospheric correction, FLAASH (fast line-of sight atmospheric analysis of spectral hypercube), was performed based on the results of emissivity calibration of the PMS data [39]. The gain parameters of GF-4, providing the calibration coefficients for 5 types, are shown in Table 2, and the bias is 0. For example, the band status 2­6­4­6­6 refers to green, red, near red band integration time, unit is millisecond (ms) [40]. The calculation is expressed as:

(1) Le =Gain ∗ DN + Bias,

where Le is the equivalent radiance at the entrance pupil of the satellite load channel, and gain and bias are the scaling coefficient gain and offset (http://www.cresda.com).

Where PMS is a sensor with full-color band on the GF4 satellite, and P1-P5 refers to bands 1 to bands 5.

#### 2.4.2. Atmospheric Correction

Atmospheric correction is the process of eliminating the radiation errors affected by the atmosphere [4,41]. FLAASH was applied in this article for atmospheric correction, using ENVI (Environment for Visualizing Images) software based on the effects of radiation calibration. The standard moderate spectral resolution atmospheric tran smittance algorithm and computer model (MODTRAN) atmospheric model and the aerosol type of imagery can be used directly [42]. FLAASH, being on the basis of MODTRAN4 + radiative transmission model, has high accuracy. The FLAASH atmospheric correction tool avoids the measurement of atmospheric parameters that are synchronized with the image imaging [43]. It can obtain the estimation of atmospheric properties based on the spectral information of the pixels on the image maintaining a high degree of reducibility in the spectral information of the features, and obtain more accurate physical model parameters of the features [44,45]. Furthermore, the application of the FLAASH model can also correct the “neighborhood effect” caused by diffuse reflection and perform spectral smoothing on spectral noise caused by artificial suppression [46].

#### 2.4.3. Geometrical Correction of Image

The geometric correction of a remote-sensing image is to correct all kinds of distortion in the process of imaging and obtain a consistent image [47]. A new image required by a map projection or graphic expression is an important link in remote-sensing image processing and the most complex and dense part of calculation [48]. In this paper, the geometric correction module built in ENVI is used for correction.

#### 2.4.4. Inversion Modeling Method

Firstly, the band combination with the highest correlation coefficient will be selected to establish the Chla concentration inversion model in the Bohai Sea. The in situ samples are first randomly divided into a modeling group and a testing group [49]. The modeling group data were applied by linear, polynomial and exponential means to obtain curve fit for the above factors and the measured Chla concentration. Finally, the model was tested using in-situ data.

All calculations were performed in the software ENVI and Python 3.7.

## 3. Result

### 3.1. Correlation Analysis of Panchromatic Multispectral Sensor (PMS) Inversion Algorithm

We collected 146 samples of which 78 were used to establish the inversion formula, and the rest were used to verify the retrieval results. The detailed analysis is as follows.

#### Sensitive Bands of Chlorophyll-A (Chla)

The spectral character of water changes with the change of Chla concentrations. With the increase of Chla concentration, the absorption near 450 nm and 660 nm increases, and it decreases near 560 nm, while the reflection value increases relatively near 700 nm [50]. The Chla concentration can be estimated based on the reflectance at different wavelengths [51].

**Figure 2:**
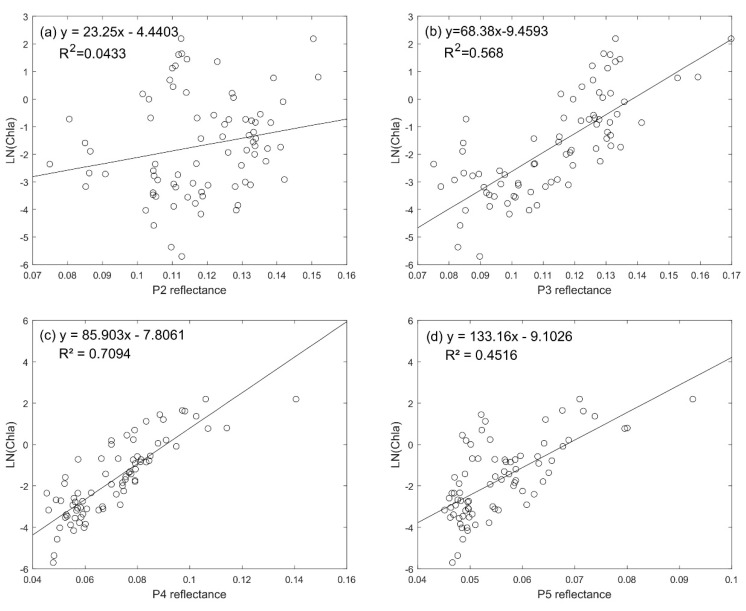
Correlation between band reflectance and sampled chlorophyll-a (Chla). (**a**–**d**): The linear relationship between the reflectance of band 2,3,4,5 and Chla concentration. The abscissa is the reflectance of a single band, and the ordinate is the natural logarithm of Chla (Ln(Chla)).

A linear fit was performed using the measured Chla concentration, based on the reflectance data in 2~5 bands, and the correlation coefficients were analyzed (Figure 2). The correlation coefficient between the single band and Chla is small with the value between 0.04 and 0.71 indicating low correlation (Figure 2). Therefore, the single band cannot be applied to perform Chla inversion. Bohai Sea is a typical type II water body and its spectral characteristics are not entirely generated by Chla, but by the combined action of CDOM, suspended sediment, and Chla [52].

### 3.2. Band Combination

Based on the single-band factor correlation analysis above, four bands (P2, P3, P4, P5) other than panchromatic band (P1) are combined flexibly to reveal their correlation with Chla [43]. Twenty band combinations with relatively high correlation with Chla concentration were applied for analysis in this study (as shown in Table 3).

**Figure 3:**
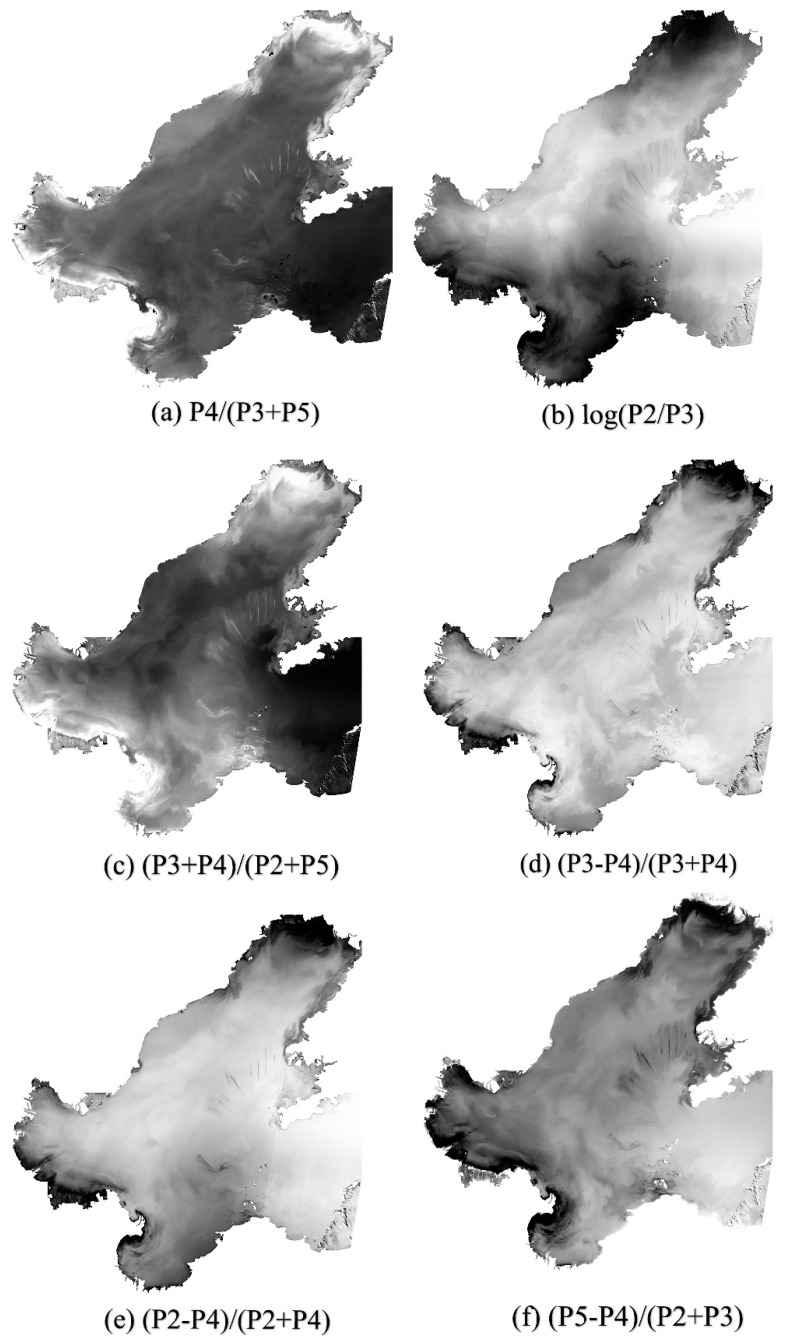
Band combined image of 2 September 2019. (**a**): The grayscale image of band combination P4/(P3 + P5);(**b**): The grayscale image of band combination log(P2/P3); (**c**): The grayscale image of band combination (P3 + P4)/(P2 + P5); (**d**): The grayscale image of band combination (P3 − P4)/(P3 + P4), (**e**): The grayscale image of band combination (P2 − P4)/(P2 + P4); (**f**): The grayscale image of band combination (P5 − P4)/(P2 + P3).

According to the correlation analysis in Table 3, six combinations with strong correlation, and large correlation coefficient, are selected for detailed analysis and interpretation. Figure 3 is the grayscale map corresponding to the combination of six bands. Different band combinations provide different information, and the degree of correlation with Chla concentration is also different.

### 3.3. Model Building

On the basis of the above analysis, linear, quadratic and exponential fittings were performed for the selected 6 band combinations and the measured Chla data, respectively, and 24 different models are fitted, as shown in Table 4. X in Table 4 is the band combination, R^2^ is the correlation coefficient and RMSE is the root-mean-square error. Secondary optimization screening was conducted again to select 6 models with high correlation coefficients in the 24 models (underlined in Table 4).

**Figure 4:**
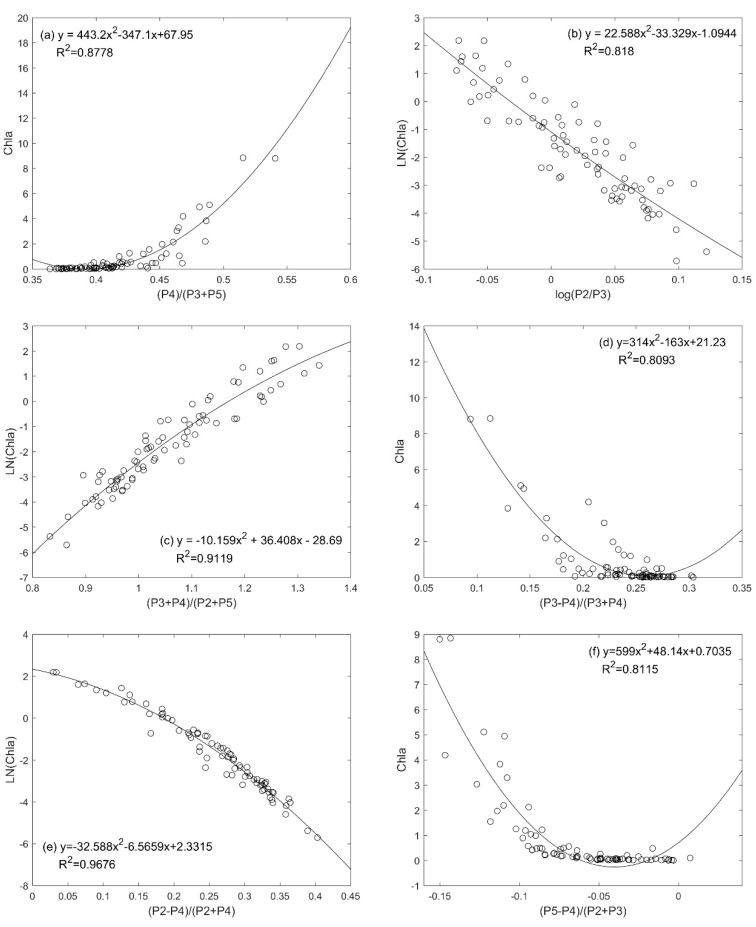
Curve fitting results of 6 models. The abscissa represents the combination ratio of band reflectivity. The ordinate in Figure 4b,c,e is the natural log of the measured data (Ln(Chla)), and the ordinate in Figure 4a,d,f is the measured data for Chla. (**a**): The correlation analysis between P4/(P3 + P5) band combination and Chla; (**b**): The correlation analysis between log(P2/P3) band combination and LN(Chla); (**c**): The correlation analysis between (P3 + P4)/(P2 + P5) band combination and LN(Chla); (**d**): The correlation analysis between (P3 – P4)/(P3 + P4) band combination and Chla; (**e**): The correlation analysis between (P2 − P4)/(P2 + P4) band combination and LN(Chla); (**f**): The correlation analysis between (P5 − P4)/(P2 + P3) band combination and Chla,

The fitting results with a high correlation coefficient of 6 models in Table 4 are shown in the Figure 4a–f correspond to the band combinations in Table 4. It can be seen from the correlation analysis in Figure 4 that the correlation coefficient of the exponential fitting form model of band combination (P2 − P4)/(P2 + P4) is the largest.

The modeling group data were applied, by linear, polynomial, and exponential models, to get curve fit for the above factors and the measured Chla concentration. The combination method with the largest correlation coefficient is selected to establish a Chla inversion model.

The band combination with the highest correlation coefficient is the fifteenth combination in Table 4, so the final model (named PMS-1) for Chla inversion in the Bohai Sea area of PMS data is finally determined as follows:

(2) $$\rho =\;\exp\left(2.3315-6.5659X-32.588X^{2}\right),$$

(3) X=(P2−P4)/(P2+P4),

where ρ is the Chla concentration, the unit is µg/L, P2 and P4 are the reflectance of the 2nd and 4th bands of PMS data.

**Figure 5:**
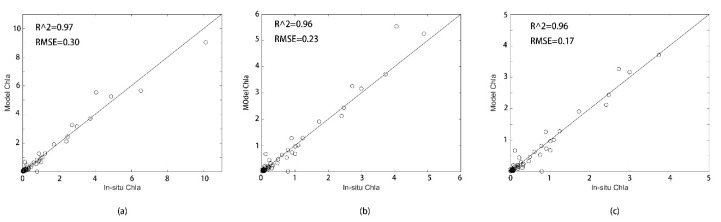
Comparison of modeled Chla concentration value and in situ Chla value, the x-coordinate is in situ Chla, and the y-coordinate is model Chla, both in units µg/L. In addition, (**a**) shows the correlation fitting of 68 test sampling points with Chla inversion simulated concentration, (**b**) shows the correlation fitting of 67 test sampling points with Chla inversion simulated concentration lower than 6 µg/L, (**c**) shows the correlation fitting of 64 test sampling points with Chla inversion simulated concentration lower than 5 µg/L.3.4. Model retrieved Chla concentration in the Bohai Sea.

Compared measurements from two sources (e.g., field and remote-sensing ones) are necessary [53]. In total, 146 in situ measurements, of which 78 were used to establish the inversion formula, and the rest 68 were used to verify the retrieval results (Figure 5). In this paper, the correlation coefficients of modeled Chla and in situ Chla were analyzed to test the significance of the above formula and showed a significant result with R^2^ = 0.97, and the critical value of the correlation coefficient r0.05 = 0.374, r0.01 = 0.478, r > r0.01. This linear relationship is considered to be significant. In Figure 5a, the concentration range of Chla is 0–11µg/L, R^2^ is 0.97, and RMSE is 0.30. In Figure 5b, the correlation analysis between the sampling point and the simulation point with Chla concentration below 6 µg/L, the R^2^ is 0.96 and RMSE is 0.23. In Figure 5c, the correlation analysis between the sampling point and the simulation point with Chla concentration below 6 µg/L, the R^2^ is 0.96 and RMSE is 0.17. 

**Figure 6:**
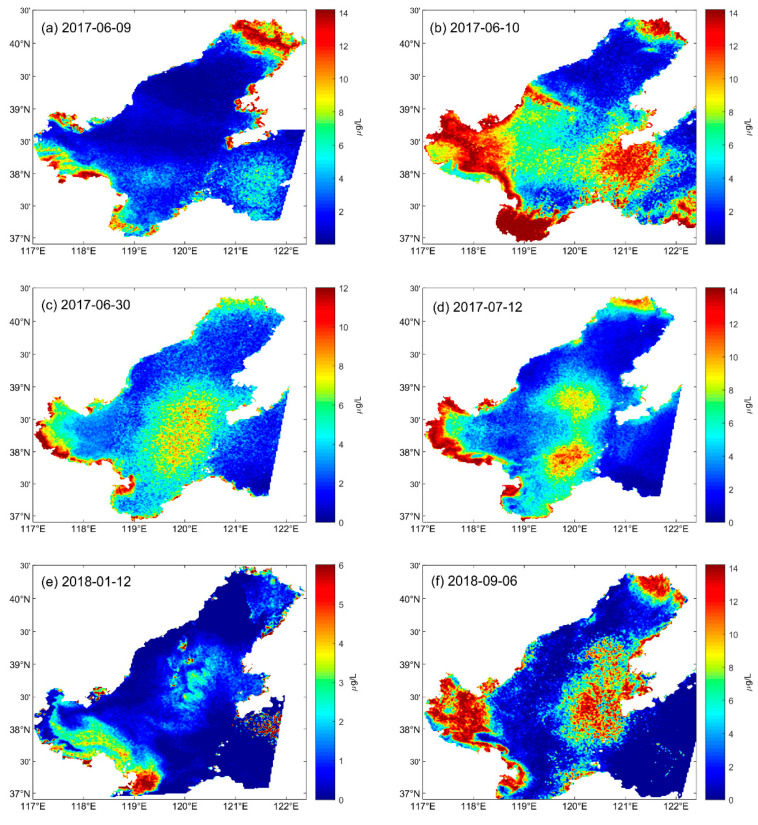
The distribution of Chla concentration in Bohai Sea. Figure 6a–f is the spatial distribution diagram of Chla concentration at different times after inversion using the empirical formula obtained, the unit is µg/L. (**a**): The image Chla inversion concentration distribution map obtained on 9 June 2017; (**b**): The image Chla inversion concentration distribution map obtained on 10 June 2017; (**c**): The image Chla inversion concentration distribution map obtained on 30 June 2017; (**d**): The image Chla inversion concentration distribution map obtained on 12 July 2017; (**e**): The image Chla inversion concentration distribution map obtained on 12 January 2018; (**f**): The image Chla inversion concentration distribution map obtained on 6 September 2018.

The distribution of Chla from June 2017 to September 2018 in the study area was retrieved (Figure 6) using the new model. Twenty PMS images obtained from the National Satellite Ocean Application Service (https://osdds.nsoas.org.cn) in China were analyzed and six PMS images were taken as examples (Figure 6). The Chla concentration in Bohai Sea is mainly below 14 µg/L and the high concentration areas are usually distributed in coastal waters, Laizhou Bay and the eastern Bohai Sea. The concentration of Chla in the Bohai Strait and northern Yellow Sea is relatively low with the value being around 5 µg/L. Furthermore, the average value of Chla concentration generally shows a trend high offshore, low in the distant sea, and the low value dominates most area of Bohai Sea. This is consistent with the actual situation and prior studies [4].

### 3.4. Details of Short-Term Change in Chla Concentration

**Figure 7:**
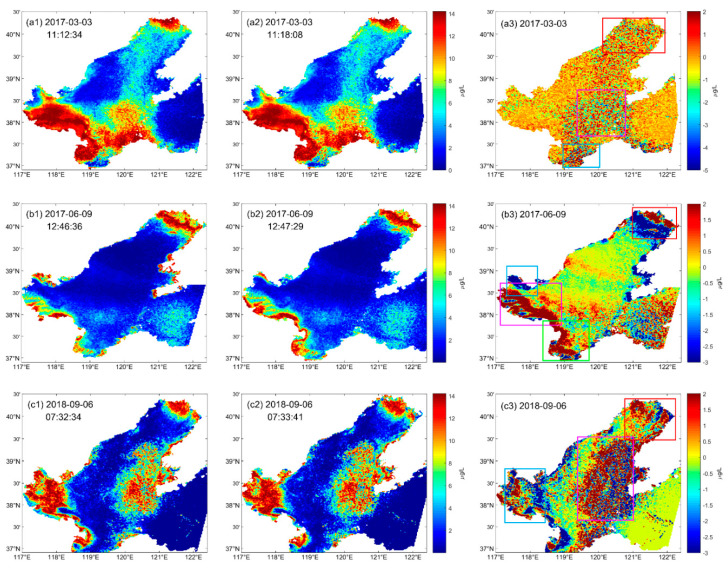
(**a1**)–(**c1**), (**a2**)–(**c2**): Chla concentration distribution at different times of the day. (**a1**)–(**a2**): the data at 11:12:34 and 11:18:08 on 3 March 2017, respectively; (**b1**)–(**b2**): the data at 12:46:36 and 12:47:29 on 9 June 2017, respectively; (**c1**)–(**c2**): the data at 07:32:34 and 07:33:41 on 6 September 2018, respectively; (**a3**)–(**c3**): changes in Chla concentration at two different times of the day. Colored squares: Chla change areas.

Details of short-term change in Chla concentration can be detected by geostationary orbit satellite GF-4. As can be seen from Figure 7a(1,2), the Chla concentration, on 3 March 2017, is less than 14 µg/L on the whole. At 11:12:34 s, the concentration of Chla was higher than 10 µg/L along the coast of Liaodong Bay (the position shown in the red square in Figure 7(a3)) and Laizhou Bay (the position shown in the blue square in Figure 7(a3)), and lower than 5 µg/L offshore of in Bohai Sea. After a few minutes, the Chla concentration changed, showing in Figure 7(a3), with the decrease in the south central part (the pink square in Figure 7(a3)) and increase in northeast part (red square in Figure 7(a3)). Furthermore, the change of Chla concentration in one minute can also been detected (Figure 7(b1–3)), and the obvious increases appear in the top of northeast part and southwest (the red, blue, pink and green square in Figure 7(b3)) coastal line. However, on 6 September 2018 (Figure 7(c1–3)), Chla concentration decreased, in southwest coastal line (the blue square in Figure 7(c3)) and increase in the northeast (the red square in Figure 7(c3)) and in the middle of the Bohai Sea (the pink square in Figure 7(c3)) in one minute, dramatically.

## 4. Discussion

### 4.1. Feasibility and Necessity of the Gaofen-4 (GF-4) PMS-1 Model in the Bohai Sea

The reflectivity of water body is far lower than that of other surface features, especially for a clear water body. Except for strong reflection in the blue and green wave bands, the absorption is obvious in the other optical wave range, and the absorption is more intense in the near-infrared wave band [15].

However, when the water is mixed with other substances, the reflection spectral curve of the water will change due to the spectral interference of other substances. Chla has unique reflection spectral characteristics, and the absorption peak appears at the wavelength of 440 nm and 678 nm, and when the content of Chla is high, the absorption valley appears near the two wavelengths [11]. Due to the weak absorption of Chla and carotene and the scattering effect of cells, reflection peaks appeared near 550 nm. For water containing algae, its spectral curve has remarkable characteristics, presenting obvious fluorescence peak within the range of 685–715 nm. The concentration of Chla will affect the location and value of the peak, since the change of Chla concentration in seawater can induce the change of water spectral characteristics [54]. Based on the change of spectral reflectance value obtained by the PMS sensor, the sensitivity of band 2–5 of PMS image to the concentration of Chla in Bohai Sea waters was analyzed.

Prior studies found that, when constructing the model for inversion of Chla concentration with characteristic bands, the accuracy of the model obtained from the combination of different bands is higher than that of the model constituted by a single band [55]. Therefore, we applied band combination to build the Chla inversion model.

GF-4 can g perform continuous observation during marine disasters, and obtain remote-sensing information at high frequency and efficiency. Because of its high spatial resolution, GF-4 has great potential for remote-sensing applications in the marine environment [56]. However, the existing conventional Chla algorithms, such as OC3, OC4, and GSM, are not suitable for GF-4 band settings [28]. Therefore, a set of inversion algorithms suitable for GF-4 satellite remote-sensing data is urgently needed. According to the reflectance value of the satellite data and the in situ Chla concentration values, a regression model was established for GF-4 PMS.

The article combines in situ data and reflectance to analyze the correlation between single-band and multi-band combinations respectively, and find the regression equation with the greatest correlation and fit. The perform error analysis on the results confirmed the feasibility of empirical models. Prior studies showed that the difference of Chla concentration was obvious at different locations in the study area, with the higher being around 14 µg/L, appears in coastal areas, and the lower value being around 3 µg/L, appears in the distant sea and the low-value region is widely distributed [16,27]. Our modeled results show a good consistency with prior studies. This is the first domestic use of GF-4 remote-sensing data in the Bohai Sea to establish a suitable model for Chla concentration, which can provide effective technical support for marine civilization construction and marine disaster investigation.

### 4.2. Factors Affecting Chla Concentration in the Bohai Sea

The change of Chla concentration in the Bohai sea is not only related to the environmental factors such as seawater temperature, salinity, illumination and nutrient salts, but also related to the vertical mixing of seawater caused by dynamic factors such as wind, flow field and tidal current [51]. The wind field and flow field (such as upwelling), the input of terrestrial materials, the feeding of zooplankton and other factors also have a direct influence on the spatial and temporal distribution of phytoplankton Chla concentration [52].

Chla distribution is subject to ocean current imaging and moves with ocean currents [57]. Along with the evolution of the coastal topography of the Bohai Sea, the cotidal time line located in the vicinity of the Bohai Sea Strait has basically shifted to the east as a whole, and the cotidal time line in most parts of the Bohai Sea has shifted counterclockwise [58]. The deflection of the line with tidal time leads to the universal advance of the full diurnal equinox tidal time in the Bohai Sea. In most of the sea areas, with no tidal point as the center along the direction of the wave rotation and propagation, the advance of tidal time–time gradually increases [59]. The Bohai sea tidal currents have two types of tidal currents, including regular semi-diurnal tidal currents and irregular semi-diurnal tidal currents, which are regular semi-diurnal tidal current in Liaodong Bay, Bohai Bay and Laizhou Bay, and irregular semi-diurnal tidal current in the central part of Bohai Sea [60]. The tides in the Bohai sea are basically irregular semi-diurnal tides with a tidal cycle of about 6 h [61]. The tidal components M2 and K1 are the most significant tidal components in the Bohai Sea. When the tide is high, the tide is southward; when the tide is low, the tide is northwards, with an average velocity of 0.5–1.0 m/s [62]. When the tidal wave moves, the distribution of Chla concentration in the sea surface layer will change.

Wind also affects ocean currents, inducing currents such as rip currents [63], thus causing changes in Chla concentrations. The northern Bohai Sea residual current velocity is smaller than other coastal current. The coastal current system not only presents certain circulation law in its own sea area, but also interacts with coastal currents in other sea areas, which has important influence on the material transport among all sea areas. The hydrodynamic analysis of three bays in the north of the Bohai Sea shows that the hydrodynamic force in the north of the Bohai Sea is mainly tidal current and the residual current is weak [57]. Therefore, the transport of Chla in the Bohai Sea mainly depends on the tidal current. In addition, the upwelling and cold vortex of wind often promote the growth of phytoplankton after a significant wind. Cyclonic vorticity caused by wind increases the distribution of surface Chla not only in time but also in space [64]. The short-term Chla change, such as changes in Chla concentration within 10 minutes, is mainly caused by ocean currents and wind. The characteristics of the average state of Chla concentration are that it is distributed in a band from the near shore to the far sea and gradually decreases from the near shore to the offshore deep sea area.

## 5. Conclusions

The GF-4 satellite, a geosynchronous orbit optical satellite with a high resolution of 50 m, was first applied to detect the Chla changing details in Bohai Sea. A newly built Chla inversion model PMS-1 for the GF-4 PMS sensor was established. The new model PMS-1 can extract Chla in the Bohai Sea well with the correlation coefficient R^2^ = 0.97, the total average relative error of 37.42% and the root-mean-square error (RMSE) of 29.61%.

The high Chla concentration is mainly located in coastal areas, such as the top of Laizhou Bay, Bohai Bay and Liaodong Bay, with the value being around 14 µg/ L. The low Chla concentration is mainly located in the Bohai Strait and northern Yellow Sea, with the value being around 5 µg/L. Generally, the mean value of Chla showed a general trend of being high in the offshore area, and low in the distant sea.

Furthermore, by taking full advantage of the continuous observation of the geostationary orbit satellite, GF-4 with a high resolution sensor PMS of 50 m, can effectively detect short-term change (changes within 10 min) in Chla concentration. The changes mainly appear at the southwest and northeast costal area as well as in the center of the Bohai Sea with the change value of around 3 µg/L. This short-term change (changes within 10 minutes) in Chla concentration is mainly caused by ocean currents and winds. Therefore, the advantage of GF-4 can be applied to make more subtle monitoring of marine environmental factors.

## Figures and Tables

**Table 1:** Band information of panchromatic multispectral sensor (PMS) on the Gaofen-4 (GF-4) satellite.

Type	Band Number	Band Range (µm)	Spatial Resolution (m)	Width (km)	Revisit Time
**Near-Infrared** (VINR)	1	0.45~0.90	50	400	20 s
	2	0.45~0.52			
	3	0.52~0.60			
	4	0.63~0.69			
	5	0.76~0.90			
**Middle Infra-red** (MWIR)	6	3.5~4.1	400		

**Table 2:** Parameters for GF-4.

PMS/Gain	P1	P2	P3	P4	P5
2,6,4,6,6	0.5395	1.0028	1.0418	0.8017	0.5655
4,16,12,16,16	0.3327	0.3803	0.3863	0.3299	0.2343
6,20,16,20,20	0.1752	0.3531	0.2750	0.2946	0.2038
6,40,30,40,40	0.1735	0.1375	0.1308	0.1171	0.0818
8,30,20,30,30	0.1288	0.1887	0.2030	0.1569	0.1084

**Table 3:** Band combination.

Band Combination (X)	Correlation Coefficient (R^2^)
(P5 − P4)/(P5 + P4)	0.68
log(P2/P3)	0.82
(P3 + P4)/(P2 + P5)	0.91
(P3 − P4)/(P3 + P4)	0.81
(P2 − P4)/(P2 + P4)	0.97
(P5 − P4)/(P2 + P3)	0.81
P5/(P3 + P5)	0.11
P5/(P4 + P5)	0.67
P5/(P2 + P4)	0.01
P5/(P3 + P4)	0.29
P5/(P2 + P3)	0.09
P5/(P2 + P5)	0.34
P4/(P3 + P5)	0.88
P4/(P2 + P3)	0.77
P4/(P4 + P5)	0.67
P4/(P3 + P4)	0.55
P4/(P2−P4)	0.66
P4/(P2−P5)	0.74
(P3 − P5)/(P3 + P5)	0.11
(P2 − P5)/(P2 + P5)	0.34

**Table 4:** 24 Models of six band combinations and the corresponding correlation coefficients (R^2^) and root-mean-square error (RMSE).

Band Combination(X)	Function	Fitting Model	R^2^	RMSE (µg/L)
(P4)/(P3 + P5)	linear	38.01X − 14.97	0.66	1.005
(P4)/(P3 + P5)	quadratic	443.2X^2^ − 347.1X + 67.95	0.88	0.6102
(P4)/(P3 + P5)	exponential	exp(−138X^2^ + 163.3X − 45.55)	0.78	0.8685
(P4)/(P3+P5)	exponential	exp(43.36X − 19.73)	0.79	0.8374
log(P2/P3)	linear	–22.08X + 1.287	0.42	1.3245
log(P2/P3)	quadratic	262.4X^2^ − 29.92X + 0.6821	0.58	1.1363
log(P2/P3)	exponential	exp(22.588X^2^ − 33.329X − 1.0944)	0.82	0.7866
log(P2/P3)	exponential	exp(–32.65X − 1.042)	0.82	0.7837
(P3 + P4)/(P2 + P5)	linear	10.11X − 9.847	0.50	1.2230
(P3 + P4)/(P2 + P5)	quadratic	44.23X^2^ − 86.04X + 41.73	0.66	1.0202
(P3 + P4)/(P2 + P5)	exponential	exp(–10.159X^2^ + 36.408X − 28.69)	0.91	0.5473
(P3 + P4)/(P2 + P5)	exponential	exp(14.32X − 16.84)	0.90	0.566
(P3 − P4)/(P3 + P4)	linear	−31.08X + 8.148	0.61	1.0893
(P3 − P4)/(P3 + P4)	quadratic	314X^2^ − 163X + 21.23	0.81	0.7623
(P3 − P4)/(P3 + P4)	exponential	exp(−42.755X^2^ − 14.195X + 4.0796)	0.58	1.1882
(P3 − P4)/(P3 + P4)	exponential	exp(–32.16X + 5.861)	0.58	1.1851
(P2 − P4)/(P2 + P4)	linear	−16.88X + 5.154	0.68	0.9836
(P2 − P4) /(P2 + P4)	quadratic	104.5X^2^ − 63.29X + 9.459	0.95	0.4051
(P2 − P4) /(P2 + P4)	exponential	exp (−32.588X^2^−6.5659X + 2.3315)	0.97	0.3317
(P2 − P4)/(P2 + P4)	exponential	exp(−21.03X + 3.673)	0.94	0.4322
(P5 − P4)/(P2 + P3)	linear	−33.52X − 1.268	0.51	1.2139
(P5 − P4)/(P2 + P3)	quadratic	599X^2^ + 48.14X + 0.7035	0.81	0.758
(P5 − P4)/(P2 + P3)	exponential	exp(131.95X^2^ − 26.229X − 4.0486)	0.81	0.8062
(P5 − P4)/(P2 + P3)	exponential	exp(−44.22X − 4.483)	0.80	0.8279
